# Respiratory virus dynamics in a tropical region: Insights from Yucatán, México (2018–2024)

**DOI:** 10.1017/S0950268825100939

**Published:** 2026-01-02

**Authors:** Marco Antonio Jiménez-Rico, David Fernando Novelo-Pérez, Claudia Isabel Puch-Magaña, Raquel Andrea Manrique-Puch, María de Lourdes Puerto-Compean, Rodrigo García-López, Ericka Nelly Pompa-Mera, Mireya Núñez-Armendáriz, Rosa Elena Sarmiento-Silva, Miriam Lugo-Tavera

**Affiliations:** 1 Laboratorio de Análisis Clínicos, Clínica de Mérida, Mexico; 2 Universidad Nacional Autonoma de México Instituto de Biotecnologia, Mexico; 3 Unidad de Investigación Médica en Inmunología e Infectología, Hospital de Infectología Dr Daniel Mendez Hernandez, Mexico; 4 Unidad de Investigación Médica en Enfermedades Infecciosas y Parasitarias, Centro Médico Nacional s.XXI. IMSS, Mexico; 5 Servicio de Infectología de Adultos, Hospital de Infectologia Dr Daniel Mendez Hernandez, Mexico; 6 Facultad De Medicina Veterinaria Y Zootecnia. Microbiología E Inmunología, Universidad Nacional Autónoma de México, Mexico

**Keywords:** Respiratory viruses, Influenza, SARS-CoV-2, Respiratory syncytial virus, Mexico, Yucatan Peninsula

## Abstract

The activity of respiratory viruses (RVs) displays large variability in tropical regions, posing challenges for public health response strategies. Data from most RVs in south-eastern Mexico remain limited, particularly in the Yucatan Peninsula, the largest tourism hub in the country. This retrospective study analyses the regional epidemiology of RVs in Merida, the largest city in the region, using laboratory test data from a local hospital (January 2018–April 2024). Test results of 143292 RVs were collected, including 121976 for SARS-CoV-2, 19355 for influenza A and B viruses, and 1961 for 17 distinct RVs. We found that non-SARS-CoV-2 RVs circulated year-round, with higher activity in autumn and spring, while SARS-CoV-2 peaked in summer and winter. Influenza A virus, respiratory syncytial virus, and influenza B virus reached their highest activity in autumn, earlier than in other regions of Mexico. Human metapneumovirus peaked during autumn-winter. Rhinovirus/enterovirus and parainfluenza showed year-round activity, with peaks in autumn and spring. Other coronaviruses were more frequent during winter-spring. In post-pandemic years (2022–2023), adenovirus outbreaks emerged, as well as an increased prevalence of non-SARS-CoV-2 RV co-infections. This study highlights the need for region-specific public health strategies, including optimized vaccination schedules, such as for influenza A virus, and enhanced diagnostic surveillance.

## Introduction

Respiratory viruses (RVs) are among the leading causes of morbidity and mortality worldwide, especially among children and other vulnerable populations. In 2021, excluding COVID-19, more than 344 million cases of lower respiratory infections and 2.18 million deaths were estimated globally [1]. Additionally, by 2021, more than 5 million deaths caused by COVID-19 were reported worldwide [2].

While the epidemiology of non-SARS-CoV-2 RVs in temperate regions between the tropics and the poles tends to follow predictable seasonal patterns (typically in winter), the situation is less predictable in tropical climates. This has been reported for viruses like influenza A virus and respiratory syncytial virus (RSV). In these regions, the timing and prevalence of RV outbreaks can vary significantly, making it more challenging to prepare and respond effectively [3, 4].

Most importantly, surveillance of RVs remains limited in most of the global south (developing) countries such as Mexico [5]. Moreover, the lack of widespread mandatory reporting of critical pathogens such as RSV and human metapneumovirus (HMPV) prevents a thorough assessment of their public health impact [6]. Most epidemiological studies on RVs carried out in Mexico focus on high-altitude regions in its central region, such as Mexico City and its metropolitan area, which has a temperate climate or the arid regions in Northern Mexico. Data from tropical areas in the south-east remain scarce, highlighting a gap in our understanding of the dynamics of RVs in these regions [7–13].

The state of Yucatan, located in the Yucatan Peninsula in south-eastern Mexico, is characterized by a warm tropical climate with high humidity [4]. Furthermore, the large amount of both national and international tourists in the Yucatan Peninsula (2.3 million visitors in 2023) [14] represents a constant entry for imported viral pathogens. However, unlike SARS-CoV-2 and influenza A virus, epidemiological data on other RVs in Yucatan remain limited compared to other regions [4, 15–19]. The lack of regional data underscores the critical need for comprehensive surveillance to better understand the circulation of RVs and design public health strategies. Epidemiological data are essential for the implementation of contingency measures and adoption of preventive strategies.

Global reports have highlighted major changes in the epidemiology of seasonal non-SARS-CoV-2 RVs during and after the COVID-19 pandemic, further emphasizing the need for updated surveillance [20–22]. The circulation of these viruses was globally reduced during pandemics. However, after the lifting of preventive measures, seasonal viruses such as influenza A and RSV reemerged, and some reports have indicated that typical seasonal patterns were disrupted. Additionally, a dramatic increase in paediatric respiratory infections has been reported [20–22].

In this study, we present epidemiological patterns of RVs in the Yucatan Peninsula, including SARS-CoV-2 and non-SARS-CoV-2 such as influenza A virus, influenza B virus, RSV, HMPV, human adenovirus (AdV), human rhinovirus/enterovirus (HRV/EV), coronaviruses (NL63, 229E, HKU-1, OC43), and parainfluenza (1, 2, 3, and 4), using data collected from a hospital laboratory in Merida, the largest city in the region. Our findings offer valuable insights into the circulation of RVs that are less commonly reported, including data from both pre- and post-COVID-19 pandemic.

## Methods

### Data collection

**Figure 1.**
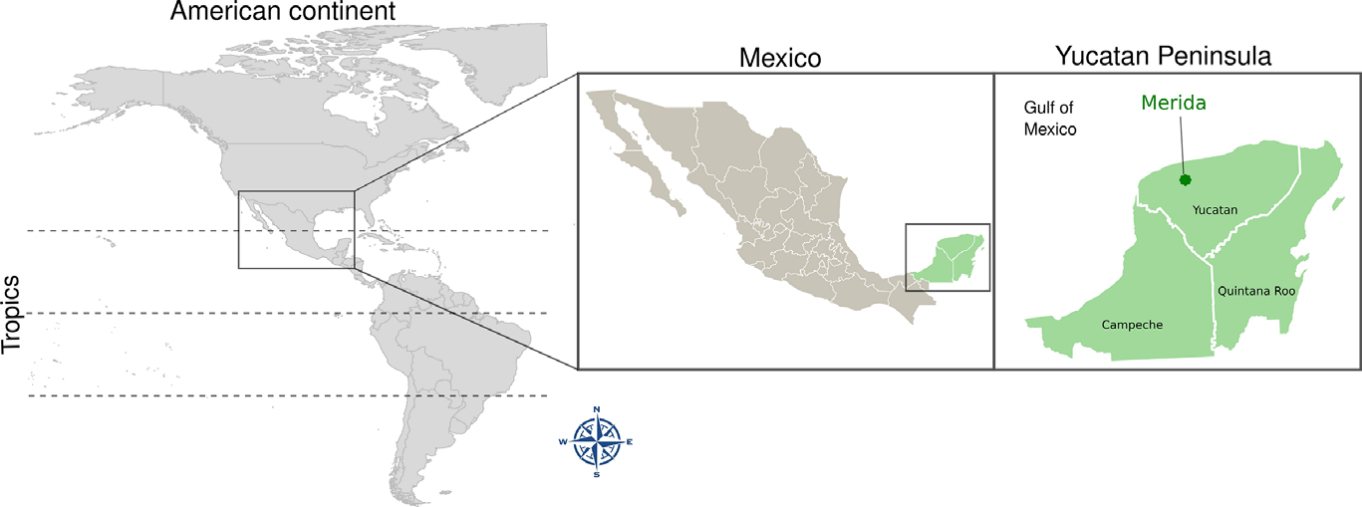
Study site. Data were collected from Clinica de Merida hospital laboratory, located in the city of Merida, Yucatan, a tropical region of Mexico. Maps were created in R using the mxmaps package [60].

This study included both positive and negative results of routine diagnostic tests for RVs previously conducted at the Clinica de Merida hospital laboratory, located in Merida, Yucatan, Mexico (Figure 1), from January 2018 to April 2024. The Clinica de Merida is a private tertiary-level medical centre, equipped with a laboratory specialized in the diagnosis of respiratory infections. In compliance with the laboratory’s privacy notice, each patient was informed that their test results may be used for research purposes. In accordance with national laws [23], this purpose is a retrospective, risk-free study for the patient, and hence, informed consent was waived. Personal data from patients (except for age, test date, and laboratory results) were fully anonymized. The study included patients of all age groups and SARS-CoV-2 tests from 2020 onwards.

Briefly, a nasal/nasopharyngeal swab was collected from each patient by the laboratory technician and investigated either through RT-PCR amplification or antigen test using standardized procedures. Results from commercial RT-PCR amplifications targeting 17 RVs (Table 1) were included. Additional sets of influenza A virus, influenza B virus, and SARS-CoV-2 confirmed with different commercial RT-PCR amplification or antigen tests were obtained as well. Diagnostic kits used are listed in Supplementary Material 1.

**Table 1.** Laboratory tests conducted for respiratory viruses diagnosis

Virus	Detection method	Total test	Positive (*n*, %)	Age median ± SD, range
Adenovirus (AdV), human rhinovirus/enterovirus (HRV/EV), respiratory syncytial virus (RSV) A/B, human metapneumovirus (HMPV), influenza A virus (H3, H1pmd2009), influenza B virus, parainfluenza virus 1, parainfluenza virus 2, parainfluenza virus 3, parainfluenza virus 4, coronavirus NL6, coronavirus 229E, coronavirus OC43, coronavirus HKU–1, SARS-CoV–2 (only by FilmArray), and bocavirus	Syndromic RT-PCR multiplex	1961: (FilmArray, *n* = 1909, Allplex Seegene (*n* = 52)	967 (49%)	54 ± 31.9, 0–104
Influenza A virus and influenza B virus	RT-PCR or antigen test	19355: 18226 antigen tests, 1119 RT-PCR	4413 (22.8%)	28 ± 23.3, 0–104
Severe acute respiratory syndrome coronavirus 2 (SARS-CoV–2)	RT-PCR or antigen test	121976 104504 antigen tests, 17472 RT-PCR	26180 (21.5%)	38 ± 19.2, 0–109

*Note:* Allplex Seegene Respiratory Panel Assay (*n* = 52) does not detect SARS-CoV-2 or human coronavirus HKU1; FilmArray Respiratory Panel Assay (*n* = 1909) does not detect human bocavirus. The complete list of diagnostic kits and their viral targets is provided in Supplementary Material 1. SD = Standard deviation.

### Statistical and epidemiological analysis

All analyses and plots were performed using the R programming language, version 4.1.3.

We compared our dataset with Mexican Health Ministry (Secretaría de Salud; SSa) reports on SARS-CoV-2 using Pearson’s correlation. A one-way ANOVA assessed differences in multiplex PCR testing frequency across years, with Tukey’s post hoc test identifying significant differences (Supplementary Material 2).

The prevalence for each RV was calculated as follows: the number of positive tests divided by the total tests performed, expressed as a percentage. Longitudinal data, including raw absolute cases and positivity of RVs, were analysed by month and presented in bar plots to visualize seasonal patterns. Density plots show the daily/monthly distribution of RVs by age, highlighting the higher frequencies. For SARS-CoV-2, influenza A and B viruses, data from both PCR and antigen tests were collated and analysed together. The prevalence of influenza A virus subtypes was analysed whenever available and presented in pie charts. Tables summarizing RV positivity by age group, month, and patient origin are presented in Supplementary Material 3.

Co-infections were defined as the positive confirmation of two or more viruses in the same sample. The prevalence of co-infections was calculated as a percentage based on total positive tests. These were illustrated in heat maps.

## Results and discussion

### Collected data

A total of 143292 laboratory test results for RVs were included in this study from 2018 up to April 2024. The median age of patients is shown in Table 1. The large majority of samples were collected for SARS-CoV-2 testing during the emergency phase of the COVID-19 pandemic (2020–2023). However, the main core set in the study was tested for multiple viruses (1961 items during 2018–2024).

### Prevalence and dynamics of non-SARS-CoV-2 RVs before and after the COVID-19 pandemic

**Figure 2.**
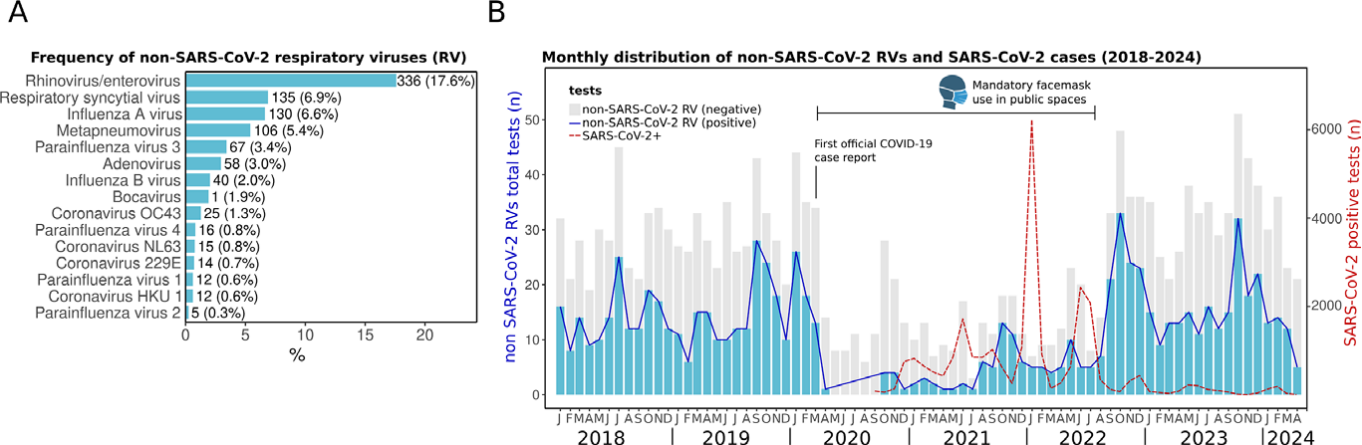
Respiratory virus prevalence from January 2018 to April 2024. (a) Frequency of non-SARS-CoV-2 respiratory viruses (RVs) in absolute (*n*) and percentages (%). (b) Monthly distribution of RVs. Bars represent the positive (blue) and negative (grey) tests (scale on the left *Y*-axis) of non-SARS-CoV-2 RVs from the multiplex panel. The red dotted line shows the monthly distribution of SARS-CoV-2 cases including RT-PCR and antigen tests (scale on the right *Y*-axis). The first official case of COVID-19 in Merida was reported on 13 March 2020. The period when face masks were mandatory in public spaces is indicated.

We observed a high positivity of non-SARS-CoV-2 RVs during the whole study period (January 2018 to April 2024). The most frequently detected were HRV/EV (17.6%), RSV (7.1%), influenza A (6.4%), and HMPV (5.3%) (Figure 2a). This distribution aligns with reports in Mexico [10, 12], reinforcing the consistent circulation of these pathogens in the country.

Before the COVID-19 pandemic, the detection of RVs was common throughout the year during the period from 2018 to early 2020 (Figure 2b). The first official COVID-19 case in the city of Merida was registered on 13 March 2020. A subsequent decline in the detection of all non-SARS-CoV-2 RVs was observed just afterwards. This same effect was reported globally, and 2020 was deemed atypical due to the near absence of other seasonal RVs, largely attributed to widespread non-pharmaceutical interventions, including mandatory lockdowns, social distancing, and travel restrictions [10, 24–26].

However, the activity of non-SARS-CoV-2 RVs increased again in late 2021 and has persisted in subsequent years, reflecting the trends observed in other countries [20, 21]. The resurgence of other RV infections has been linked to the relaxation of preventive measures. For instance, in Merida, the use of face masks in public spaces remained mandatory until September 2022 [27]. After it was lifted, a large surge of non-SARS-CoV-2 RVs was observed (Figure 2b).

### Higher detection of non-SARS-CoV-2 RVs in children

**Figure 3.**
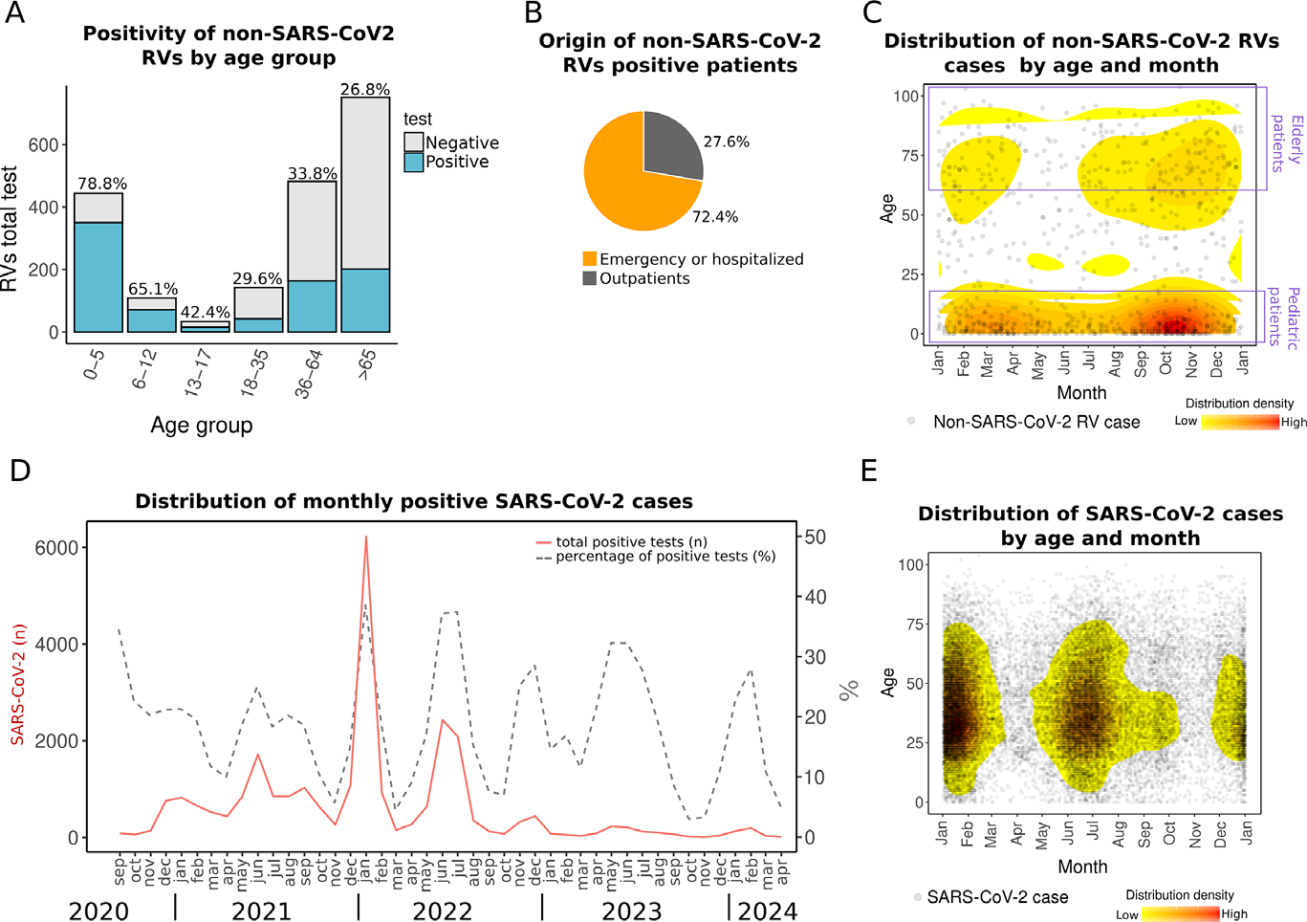
Distribution of non-SARS-CoV-2 RV and SARS-CoV-2 cases. (a) Non-SARS-CoV-2 RVs by age group. The percentage above the bars indicates RV positivity. (b) Pie chart of the distribution of RV-positive patients by origin: emergency department or hospitalized and outpatient. (c) The density plot shows all non-SARS-CoV-2 RV cases (2018–April 2024) distributed by day and month of the year (*X*-axis) versus patient age (*Y*-axis). (d) Monthly SARS-CoV-2 distribution showing total cases detected (*n*) (left *Y*-axis) and percentage (right *Y*-axis) from 2020 to April 2024. (e) Density plot of SARS-CoV-2 cases by day and month of any year versus patient age. The red colour in density plots indicates relatively higher density.

In the present study, the highest positivity for non-SARS-CoV-2 RVs was observed in children under 5 years of age (78.8%). Additionally, most positive samples (69.7%) were collected from patients who required emergency care or hospitalization (Figure 3a,b, Supplementary Material 3), reflecting the highest burden of respiratory infections in paediatric populations. While some RVs typically cause common colds, others pose a higher risk for patients. For example, HRV, which was the most prevalent in the dataset, has been associated with respiratory distress and asthma in Mexican children [28].

### Seasonality of non-SARS-CoV-2 RVs

We observed a seasonal pattern in non-SARS-CoV-2 RV detections. Although these viruses circulated year-round during 2018–2024, they were primarily concentrated in autumn, from September to November, particularly in October, and in spring, from February to April (Figure 3c).

The seasonal pattern in Yucatan contrasts with other regions of Mexico, where studies have reported higher RV prevalence during winter [11, 28]. Pre-pandemic studies in Mexico City and other states found increased RV detection in winter months, including 2004–2006 [12], December–March 2014 [29], and November 2014–2015 [29]. Post-SARS-CoV-2, a study in Mexico City observed a rise in RV cases starting mid-October 2021, peaking in January 2022 [10]. The earlier increase in RV activity observed in this study compared to other regions of Mexico may be influenced by the country’s geographical diversity. Yucatan’s tropical warm and sub-humid climate shapes its seasonality [4], while its status as a major tourist destination with significant national and international mobility, including migratory transit given its proximity to the border with Central America, likely contributes to the circulation patterns of RVs [30–32]. Genomic surveillance has highlighted the Yucatan Peninsula as a gateway for SARS-CoV-2 variants entering Mexico, and whole-genome sequencing of other RVs could further help trace introductions and diversification associated with human mobility [17, 18].

Since some of these previous studies in Mexico do not account for the same RVs analysed in this study, comparison may be difficult. While we found that non-SARS-CoV-2 viruses tend to cluster primarily in autumn and spring, each virus showed distinct seasonal activity, which is further discussed below.

### Seasonality of SARS-CoV-2

In contrast to non-SARS-CoV-2 RVs seasonality, peaks of SARS-CoV-2 display a dual seasonality, with surges occurring during summer (May–July) and winter (December–February) from 2021 to 2024. Although COVID-19 testing decreased in 2023 and 2024 compared to 2021 and 2022, the trend and seasonality still hold when analysing the monthly positivity rates (Figure 3d,e). Such seasonal patterns suggest a dynamic in which non-SARS-CoV-2 RVs and SARS-CoV-2 alternate niche dominance in the region throughout the year.

It is still debated whether SARS-CoV-2 has transitioned into an endemic phase [33–35]. Although the WHO has declared the end of the global COVID-19 emergency phase [36], SARS-CoV-2 continues to circulate worldwide. Additionally, endemism does not imply stability. As immunity declines and the virus evolves, new variants with different transmission and immune escape properties emerge, potentially affecting the disease burden [35]. Continued surveillance, preventive measures, sustained diagnostics, and vaccination, along with public health initiatives, are crucial for managing the impacts of SARS-CoV-2.

### Influenza A virus

Compared to other non-SARS-CoV-2 RVs, influenza virus is monitored more intensively in Mexico [37] and has been studied more extensively in Yucatan, with epidemiological reports spanning from 2009 through 2018 [4, 15, 16]. In this study, the first to analyse influenza A virus cases in Yucatan during the post-COVID-19 pandemic period, we cover from 2018 through 2024.

**Figure 4.**
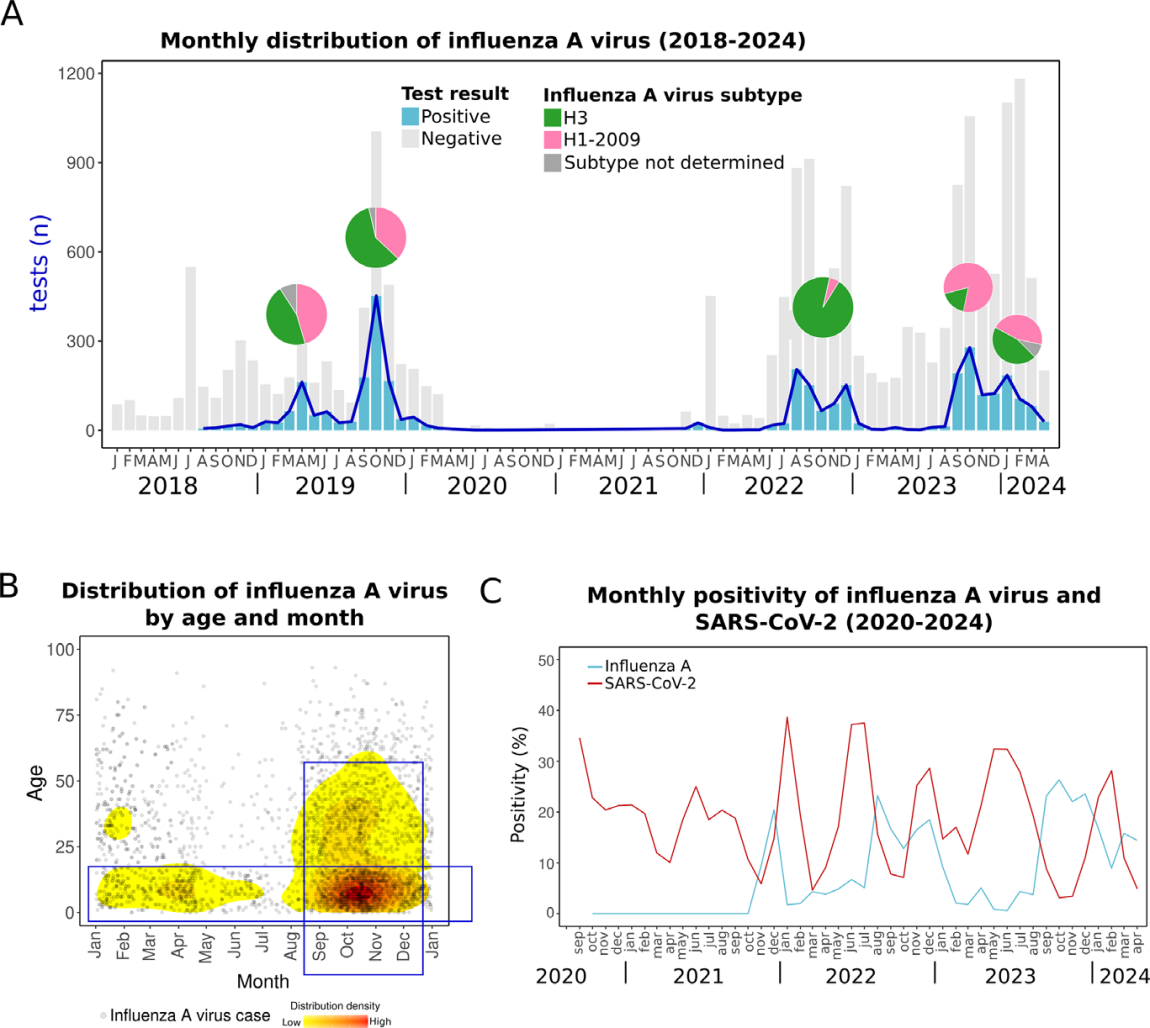
Influenza A virus cases. (a) Monthly distribution from 2018 to early 2024. Bars indicate total positive (blue) and negative (grey) tests. Pie charts over the peaks indicate the proportion of influenza A virus subtypes in each period. (b) The density plot shows all influenza A virus cases distributed by day and month and age. The red colour indicates relatively higher density. (c) Monthly positivity of influenza A virus and SARS-CoV-2.

Our findings show that influenza A virus peaks have varied annually, aligning with typical patterns of tropical regions [7, 38]. Before the COVID-19 pandemic, two peaks occurred in 2019: one in April and another in October, involving H3 and H1pdm09 subtypes, which typically alternate dominance annually. After 2 years of pandemic-related disruptions, influenza A virus reemerged in 2022 with peaks in August and December, primarily driven by the H3 subtype. Subsequent peaks occurred in October 2023, dominated by H1pdm09, and in January 2024, when both subtypes were circulating (Figure 4a).

Despite this variability, influenza A virus cases clustered during the autumn season, particularly in September–October (Figure 4b). This seasonal pattern is similar to the previous report of higher detection of influenza A virus in autumn (September) in Yucatan during 2010–2014 [4] and contrasts with seasons observed in most other Mexican states, where influenza A virus typically peaks later in winter [4].

The seasonal pattern of influenza A virus in Yucatan highlights the need to adjust vaccination timing. While current schedules follow national calendars, with vaccines administered in October and November, influenza activity in Yucatan often begins earlier. Aligning vaccination efforts with this earlier onset could improve protection and reduce influenza’s impact. The WHO recommends earlier vaccination in tropical regions, similar to the guidelines for Central America [39]. Implementing this in Yucatan would allow more time for immunity to develop before the peak season. This issue, recognized before the COVID-19 pandemic [4, 16], remains a critical concern for the region.

Influenza A virus can cause mild to severe illness and, in some cases, death. Alongside influenza A, SARS-CoV-2 is also circulating, causing similar symptoms and outcomes [40]. Our data show that throughout the year, the population in Yucatan is consistently exposed to both viruses (Figure 4c). Although the peaks of influenza A virus and SARS-CoV-2 do not appear to overlap in the last years (2023–2024), both viruses can coexist in the context of a syndemic pattern, especially during the transitional periods between their peaks. Since the treatment differs, rapid and differential diagnosis is crucial [40, 41].

### Respiratory syncytial virus

RSV is the leading cause of hospitalization and mortality in young children and a significant aetiological agent of respiratory diseases in adults [42, 43]. In this study, RSV from either subtypes A or B was the second most common non-SARS-CoV-2 RV, and detection rates varied across age groups and seasons. Most RSV detections (74.1%) occurred in children ≤5 years of age, among whom the positivity rate reached 22.5%. Notably, 78% of these paediatric cases required emergency care or hospitalization (Supplementary Material 3). RSV can infect individuals of all ages, but complications are more common at both extremes of life. In Mexico, RSV is the most common virus in community-acquired pneumonia [12], and it is linked to high mortality rates in children under 5 years old [44], leading the cause of hospitalizations and deaths from acute lower respiratory infections [45]. Factors that increase the risk of severe infection in children include prolonged hospitalization, low birth weight, premature birth (<37 weeks), and immunocompromise [46]. High-risk adults for severe RSV include those with haematological cancers, post-transplant patients on immunosuppressants, people with HIV, individuals over 60 years in nursing homes, and patients with asthma or chronic obstructive pulmonary disease [46].

**Figure 5.**
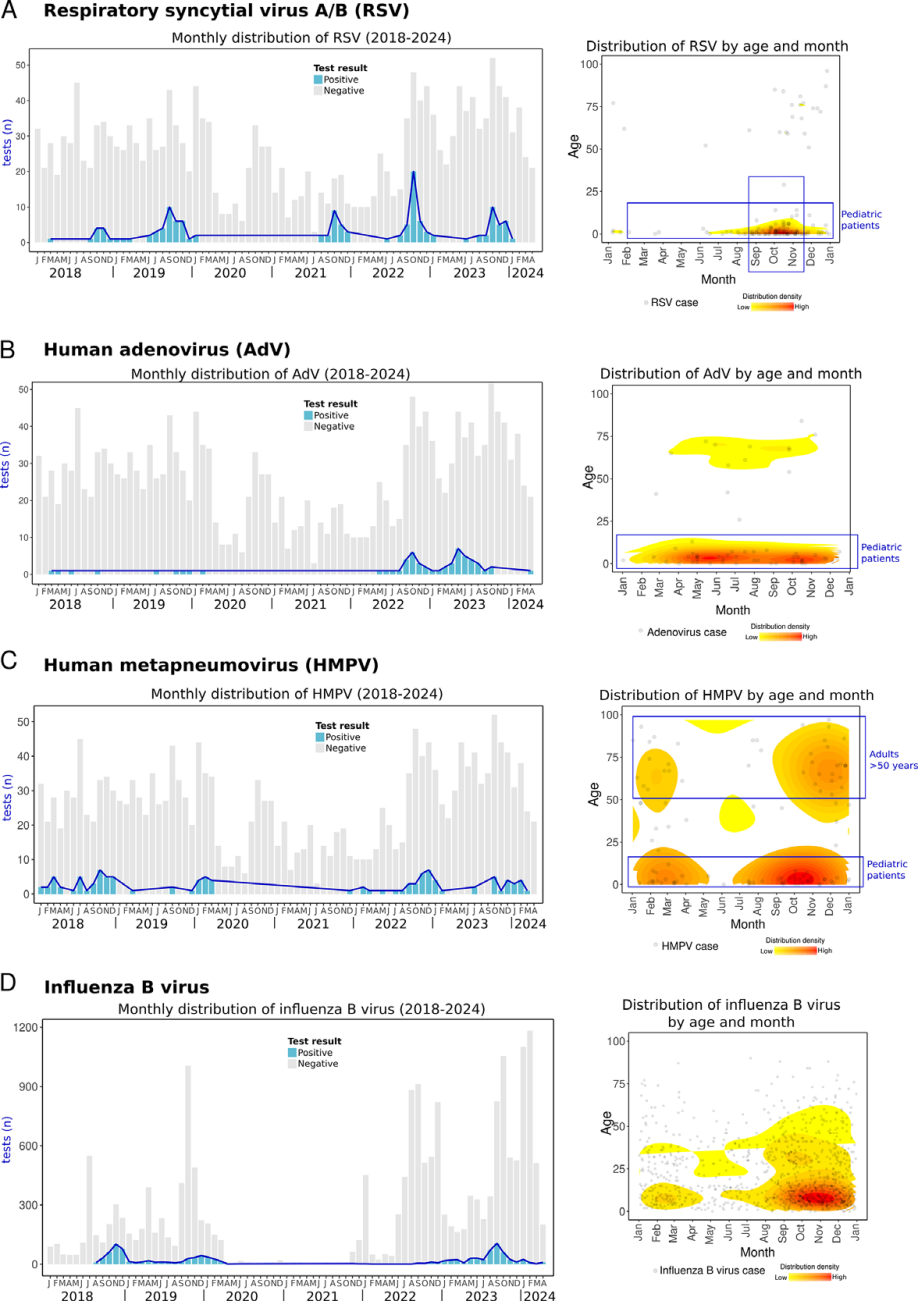
Respiratory syncytial virus A/B (RSV), adenovirus (AdV), human metapneumovirus (HMPV), and influenza B virus cases. Left side: monthly distribution from 2018 to April 2024. Bars indicate total positive (blue) and negative (grey) tests. Right side: the density plot shows all cases of each RV distributed by day/month and age (*Y*-axis). The red colour indicates relatively higher density. (a) Respiratory syncytial virus, (b) adenovirus, (c) human metapneumovirus, and (d) influenza B virus.

We observed a seasonal pattern of RSV, with cases rising at the end of summer, in August, and peaking in autumn, particularly in October, with over 40% positivity (Figure 5a). Among children ≤5 years of age, positivity reached 52–100% during these peak months (Figure 5b, Supplementary Material 3). Notably, this seasonal trend has remained consistent both before (2018–2019) and after the COVID-19 pandemic (2021–2023). The observed seasonality in Yucatan differs from patterns reported in other parts of Mexico, such as Mexico City and San Luis Potosi, where RSV activity peaks during late autumn and early winter, primarily in November and December [11, 42, 47]. These findings suggest that the RSV season in Yucatan begins earlier than in other regions in Mexico. Moreover, this agrees with previous research showing that the incidence of bronchiolitis, a complication of RSV, also increases earlier in the Yucatan Peninsula compared to other Mexican states [3].

Several factors may influence the seasonality of RSV in Yucatan. The tropical temperature and humidity of the region likely play a crucial role in modulating RSV transmission [8, 48]. In addition, air travel has been shown to shape RSV spread [30, 32], highlighting its significance in the Yucatan peninsula due to the influx of national and international tourists throughout the year. The movement of people through air travel can contribute to the geographical mixing of viral lineages, potentially impacting local transmission dynamics [30, 32]. Additionally, the onset of RSV detections generally coincides with the end of summer vacation and the reopening of schools in late August, which may facilitate transmission among children [3]. Although RSV detection has been documented in the region since 1999 [19], this is the first report of its longitudinal circulation, revealing seasonal patterns over 6 years.

The importance of RSV infection as a major public health issue highlights the urgent need for preventive measures in Mexico [45]. A recent interdisciplinary consensus led by Mexican experts in 2025 provided updated recommendations on the epidemiology, risk groups, diagnostic methods, and prevention of RSV infection in the country, including the use of monoclonal antibodies [46]. The epidemiological insights from Yucatan provided by this study are valuable, as they contribute to guiding local preventive strategies.

This surveillance data are also crucial for medical personnel, contributing to improving the diagnosis and treatment of respiratory infections in the region [46]. During the RSV season, targeted differential diagnoses are especially useful, given that influenza or other RVs may also be circulating. Multiplex PCR tests, which accurately identify RSV, influenza, and SARS-CoV-2, are approved by Mexico’s regulatory health authorities [49]. Rapid antigen detection tests for RSV can also be practical, particularly for smaller clinics without access to PCR testing.

### Adenovirus peaks after COVID-19 pandemic

AdV, typically associated with mild respiratory symptoms, can lead to severe illness in susceptible individuals [50]. Our data show that prior to the COVID-19 pandemic (2018–2019), detections of AdV were infrequent (~1% of positivity). However, two notable post-pandemic peaks were observed in autumn 2022 and in summer 2023, with positivity rates increasing up to 15% (Figure 5b).

Similarly, during the same post-pandemic periods, an increase in severe AdV infections has been reported in Colombia, USA, and South Korea, some of whom required hospitalization, intensive care or were even dead [51–53]. In our study, 48.3% of AdV cases occurred in hospitalized patients or individuals seeking care at emergency departments (Supplementary Material 3). Notably, 71.4% of these cases were children under 5 years of age (Figure 5b), reflecting the significant impact of AdV on paediatric populations.

The effects of the COVID-19 pandemic likely contributed to an increase in AdV infections. Prolonged isolation measures reduced the exposure of children to microorganisms and viruses, disrupting the natural immune stimulation required at early stages of life, a concept that has been defined as immunity gap [21, 51, 54]. As restrictions were lifted, children with limited prior exposure became more susceptible to infections. These may facilitate the wider spread of AdV serotypes that circulated at low levels in pre-pandemic years or that were not previously present in the region, which may have been introduced as global mobility rebounded after the pandemic [24, 30, 32, 55].

### Other respiratory viruses

In this study, HMPV cases were mostly detected in children ≤5 years of age (45.2% of HMPV cases), of which 75% required emergency care or hospitalization. Most HMPV cases occurred during autumn and winter (Figure 5c, Supplementary Material 3). This contrasts with the only report of HMPV in Merida, Yucatan, in 1999–2002, where the peak occurred during summer [19]. These variations in HMPV circulation underscore the need for ongoing and sustained surveillance, as HMPV has been associated with severe acute respiratory infections in Mexican children [13].

Influenza B virus activity before the COVID-19 pandemic was observed in autumn-winter in both 2018 and 2019. Cases were not observed during 2020–2021, and activity remained minimal during 2022. However, influenza B virus resurged in 2023, with cases emerging in July and peaking in autumn (September) (Figure 5d).

**Figure 6.**
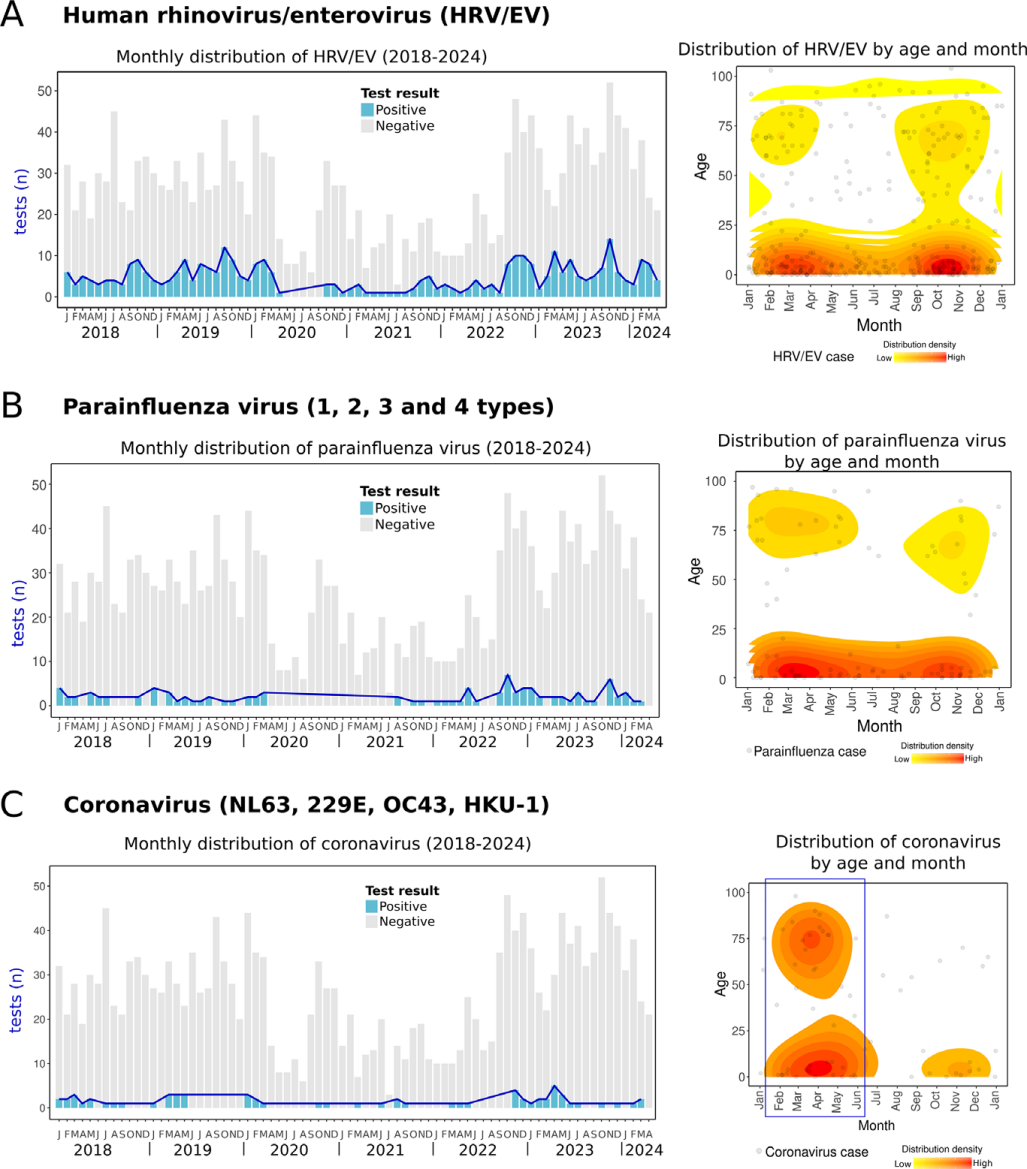
Human rhinovirus/enterovirus, parainfluenza virus, and coronaviruses cases. Left side: monthly distribution from 2018 to April 2024. Bars indicate total positive (blue) and negative (grey) tests. Right side: the density plot shows all cases of each RV distributed by day/month and age (*Y*-axis). The red colour indicates relatively higher density. (a) Human rhinovirus/enterovirus, (b) parainfluenza viruses (1,2,3, and 4 types), and (c) coronaviruses (NL63, 229E, OC43, HKU-1).

HRV/EV was one of the most frequent RVs and circulated continuously throughout the year. However, cases tended to cluster during two main periods: between winter and spring (January–May) and during autumn (September to November) (Figure 6a). Similarly, parainfluenza viruses, including four types (1–4), were also detected year-round, with two clusters observed in spring and autumn (Figure 6b). Parainfluenza virus 3 was the most prevalent type (67.7% of parainfluenza cases). Coronaviruses (NL63, OC43, 229E, and HKU-1) were less frequently detected but primarily clustered during the spring and winter months (Figure 6c). One case of bocavirus was detected in a 3-year-old child in March 2024. Since bocavirus detection was incorporated into the diagnostic panel in late 2023, its seasonality could not be determined due to the lack of data from previous years. To the author’s knowledge, no reports of HRV/EV, parainfluenza viruses, bocavirus, or non-SARS coronaviruses have been documented in the region.

### Co-infections

**Figure 7.**
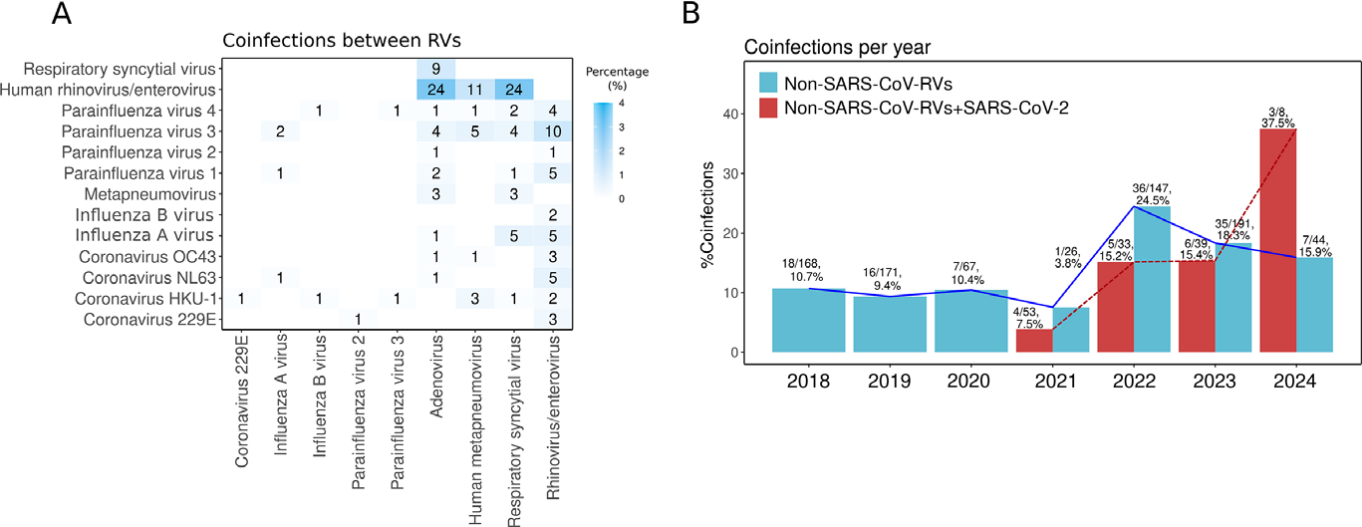
Co-infections. (a) Heat map showing the frequency of respiratory virus (RV) co-infections. Numbers inside the boxes indicate the total samples, and the gradient of blue colour represents the percentage. (b) Co-infection percentage per year between non-SARS-CoV-2 RVs (blue bars) and between SARS-CoV-2 with any RV (red bars).

Different RVs can co-circulate simultaneously and infect the same host, leading to co-infections [56]. In this study, the most prevalent co-infections were HRV/EV-RSV (2.9%), HRV/EV-AdV (2.9%), and HRV/EV-HMPV (1.3%) (Figure 7a). This distribution is similar to other reports from Mexico [10, 12].

Co-infections among non-SARS-CoV-2 RVs increased after the COVID-19 pandemic, rising from ~10% in 2018–2019 to 24.5% in 2022 and 18.3% in 2023 (Figure 7b). This trend may result from changes in RV circulation, immune dynamics, and viral evolution. As pandemic restrictions were lifted, simultaneous RV circulation increased exposure and co-infection risks. The immunity gap from reduced pathogen exposure during lockdowns likely heightened susceptibility, especially in early stages of life [21, 54]. Additionally, the genetic variability and rapid adaptation of RNA viruses may have contributed to their continued circulation [56].

Co-infections of RVs with SARS-CoV-2 were rare in 2020–2021 (<8%) but increased to 15% in 2022–2023 and over 30% in 2024. Alongside the resurgence of non-SARS-CoV-2 RVs, this increase in co-infections may be related to the evolution of SARS-CoV-2, with variants like Omicron (BA.4/BA.5) suppressing innate immunity [57]. Immune interference occurs when the immune response to one virus impacts the replication of another virus in the same host, either enhancing (synergistic) or inhibiting (antagonistic) its replication [56, 58]. The suppression of innate immunity by newer SARS-CoV-2 variants may reduce this interference, promoting greater co-circulation of RVs and co-infections.

Continuing this research is crucial for understanding how co-infections affect disease severity. Some studies indicate that co-infection with specific RVs results in greater severity, while others report the opposite [56]. Improving the surveillance of RVs and their co-infections will help monitor their role in disease severity within populations.

## Limitations

The data for this study were obtained from the laboratory of a single hospital, though in the most populated area in the region. However, the significant correlation between our dataset and the official epidemiological reports of SARS-CoV-2 suggests that these results are representative of broader trends in the region and may reflect the wider epidemiological situation for other viruses not reported by the Health Ministry (SSa) (Supplementary Material 2).

Additionally, no clinical information was available from the patients, and only data regarding their source were provided (emergency/hospital or outpatient). For outpatient cases or those who visited the emergency department, it was not known whether they were subsequently hospitalized. This limitation hinders a more comprehensive analysis of infection severity and specific risk factors, limiting our ability to adequately assess the relationship between virus prevalence and disease severity across different patient groups.

This study does not consider bacterial respiratory infections, as we focused exclusively on viruses. For instance, it is known that exposure to influenza A virus, RSV, and HMPV increases the risk of *Streptococcus pneumoniae* infection [59], showing virus–bacteria interactions are also clinically relevant and require more investigation.

## Conclusion

This is the first epidemiological report that provides seasonality data on multiple RVs in the Yucatan Peninsula, Mexico, offering valuable insights into the circulation of viruses beyond those officially reported by health authorities, including RSV, AdV, and HMPV. Although a few studies were conducted in the country, our research showed that the epidemiology of RVs in this tropical region of Mexico differs from that in other states, most noticeably in the months of higher circulation. Notably, peaks of certain RVs, such as RSV and influenza A virus, tend to occur earlier in this region than the traditional winter season observed in other regions.

Additionally, this study adds to the evidence indicating that current influenza A vaccination campaigns in Yucatan lag behind the regional start of the season, potentially limiting immunization effectiveness. Although this issue has been highlighted in previous studies, no significant changes have been made to the vaccination schedule.

The identification of RSV as a major contributor to paediatric respiratory infections and hospitalizations underscores the urgent need to consider its inclusion in future immunization and preventive strategies, including vaccination and use of monoclonal antibodies. Understanding the seasonality of these viruses is critical to refining the timing and scope of such interventions, which could significantly reduce morbidity and mortality in vulnerable populations.

Overall, the emergence and circulation of RVs and their co-infections underscore the importance of strengthening regional diagnostic and surveillance strategies. Early identification through molecular and rapid tests for differential diagnosis between respiratory infections is essential for improving public health responses. Furthermore, improved surveillance and customized preventive measures, including timely vaccination, are necessary to mitigate the risks posed by RVs, particularly for at-risk populations such as children under 5 years of age and the elderly.

## Supporting information

10.1017/S0950268825100939.sm001 Jiménez-Rico et al. supplementary material Jiménez-Rico et al. supplementary material

## Data Availability

The data that support the findings of this study are available upon reasonable request.

## Supplementary material

The supplementary material for this article can be found at http://doi.org/10.1017/S0950268825100939.
