# Supplementary material for: Respiratory virus dynamics in a tropical region: Insights from Yucatán, México (2018–2024)
Source: Epidemiol Infect. 2026 Jan 2;154:e7. doi: 10.1017/S0950268825100939 (PMC12813726; doi:10.1017/S0950268825100939)
Supplement: Jiménez-Rico et al. supplementary material [file S0950268825100939sup001.zip › Supplementary material 2.docx]

**SUPPLEMENTARY MATERIAL 2**

**Correlation of data vs official epidemiological reports.**

We compared our dataset with epidemiological reports from the Mexican Health Ministry (Secretaría de Salud; SSa), specifically focusing on SARS-CoV-2 due to the active surveillance and public availability of data for this virus. We used weekly COVID-19 cases from the city of Merida obtained from the SSa [(](about:blank)downloaded from [https://datos.covid-19.conacyt.mx](https://datos.covid-19.conacyt.mx/#DownZCSV) in October 2024[)](https://paperpile.com/c/GKAq2B/Rdse), from February 26, 2020, to June 4, 2023. Pearson’s correlation was used to compare weekly cases between both datasets. Our analysis demonstrates a strong correlation between the weekly SARS-CoV-2 cases in both datasets. Notably, there was a clear alignment of COVID-19 waves, indicating a significant concurrence in the temporal patterns of cases (Figure S1). Government reports lack publicly available surveillance data on other relevant RVs in the state of Yucatan, with limited or absent data on their prevalence. This match suggests that our dataset offers valuable insights into the circulation of other RVs that are not fully monitored by local health authorities.

**
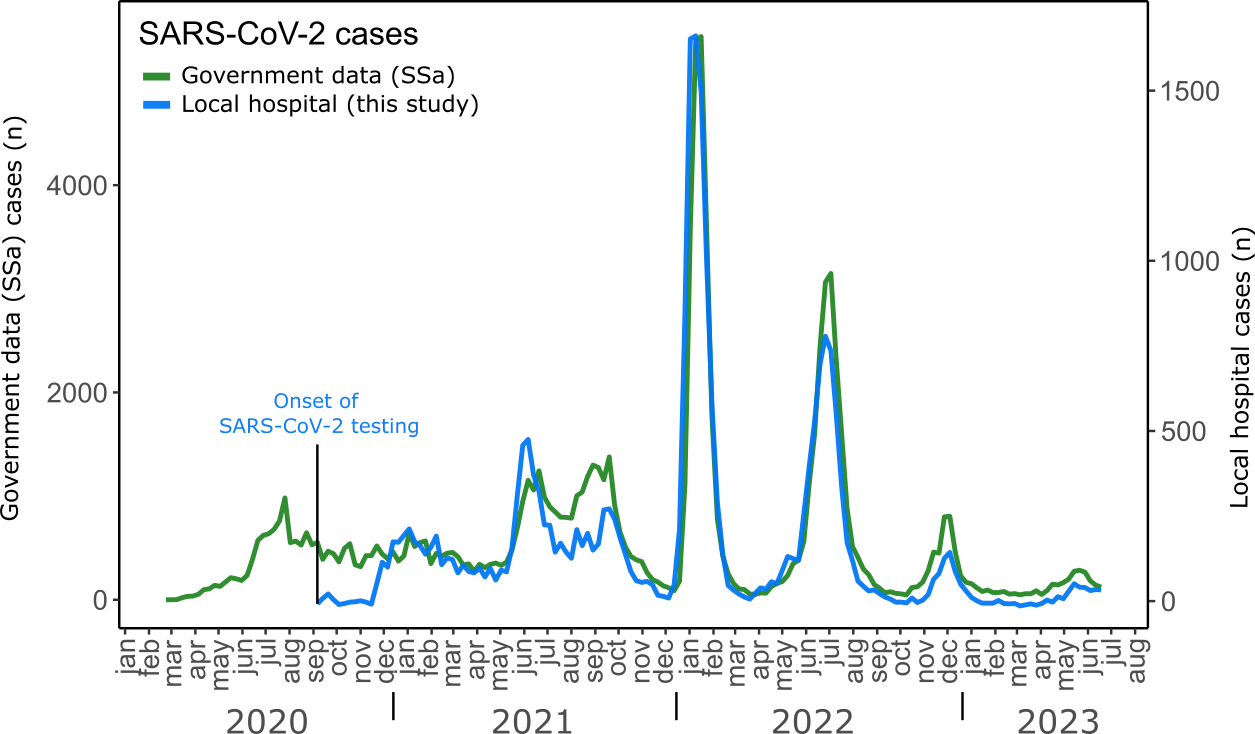
**

**Figure S1.** Comparison of SARS-CoV-2 cases: Clinica de Merida data (local hospital in this study) vs government data (SSa). Source: [COVID-19 Tablero México.” COVID - 19 Tablero México. Accessed October 25, 2024.](http://paperpile.com/b/GKAq2B/Rdse) <https://datos.covid-19.conacyt.mx/#DownZCSV>[.](http://paperpile.com/b/GKAq2B/Rdse)

**RVs PCR multiplex tests.**

The total number and average monthly number of multiplex PCR tests conducted for the diagnosis of RVs during the years 2018 and 2019 are comparable to those performed in 2022 and 2023, suggesting that comparisons in this study between pre and post-pandemic periods can provide valuable insights into the dynamics of RV epidemiology (Figure S2).

**
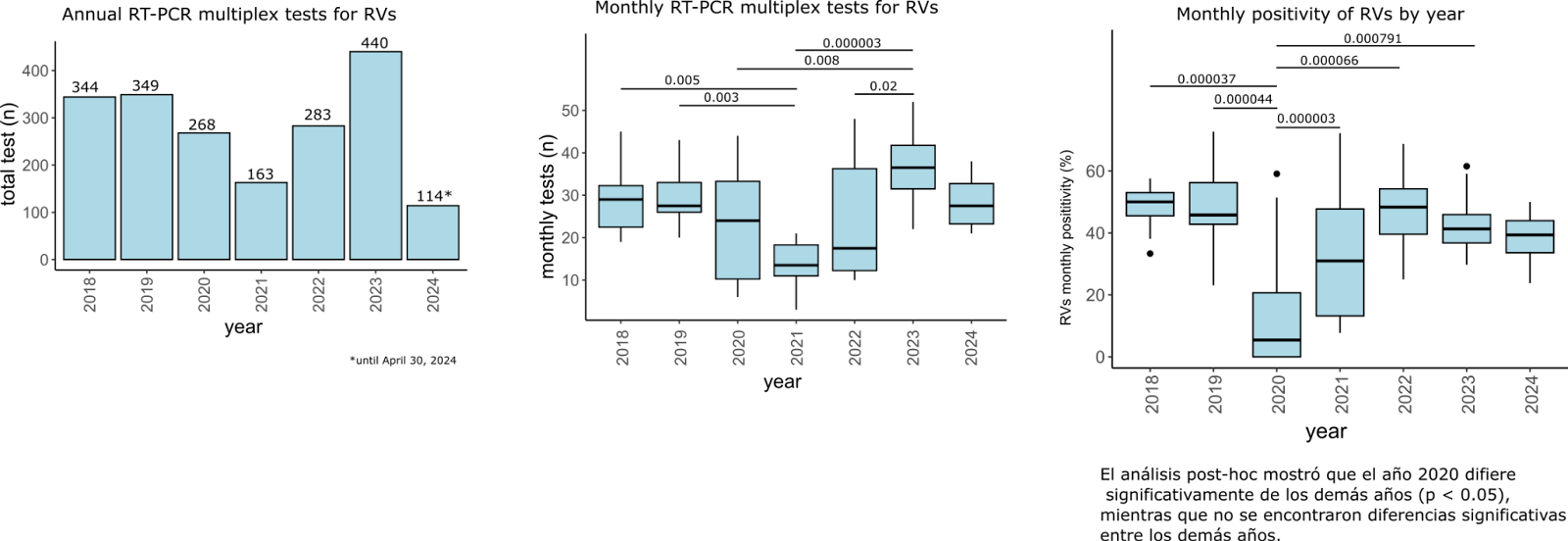
**

**Figure S2. RVs Multiplex PCR.** (A) Bars represent the total number of RV tests performed annually. (B) Boxplots display the monthly median number of tests for each year. (C) Boxplots show the monthly distribution of positive tests carried out each year. P-values with < 0.05 significance from Tukey’s post hoc test are indicated between each pair of boxes.
